# Longitudinal white matter network reorganization after rTMS in first-episode schizophrenia: associations with sleep improvement

**DOI:** 10.3389/fnins.2026.1946591

**Published:** 2026-10-01

**Authors:** Yan Li, Wenming Liu, Runzhu Sun, Yuanqiang Zhu, Min Cai, Wei Zhao

**Affiliations:** 1 The Second Affiliated Hospital of Xi'an Medical University, Xi'an, China; 2 Department of Psychiatry, Xijing Hospital, The Fourth Military Medical University, Xi'an, China; 3 Department of Radiology, Xijing Hospital, The Fourth Military Medical University, Xi'an, China; 4 Department of Emergency, Xijing Hospital, The Fourth Military Medical University, Xi'an, China; 5 Department of General Surgery, Xijing Hospital, The Fourth Military Medical University, Xi'an, China

**Keywords:** diffusion tensor imaging, first-episode schizophrenia, repetitive transcranial magnetic stimulation, rich-club organization, sleep disturbances, white matter network

## Abstract

**Background:**

Sleep disturbances are highly prevalent in first-episode schizophrenia and are associated with poor clinical and functional outcomes. Although repetitive transcranial magnetic stimulation (rTMS) has shown therapeutic potential for insomnia and schizophrenia, its efficacy for sleep disturbances in first-episode schizophrenia remains insufficiently characterized, and the underlying white matter network mechanisms remain unclear. This study aimed to determine whether active rTMS improves sleep quality compared with sham stimulation and whether longitudinal white matter network reorganization is associated with treatment-related sleep improvement.

**Methods:**

One hundred patients with first-episode schizophrenia were included in the final analysis and randomly assigned to receive active rTMS or sham stimulation over an 8-week intervention period. Subjective sleep quality was assessed using the Pittsburgh Sleep Quality Index (PSQI), and objective sleep parameters were measured using wrist actigraphy. Diffusion tensor imaging was acquired at baseline and after treatment to construct individual white matter structural networks. Graph-theoretical metrics, nodal efficiency, and rich-club organization were examined. Linear mixed-effects models were used to evaluate group-by-time interaction effects, and correlation analyses were performed to examine associations between network changes and sleep improvement.

**Results:**

Compared with sham stimulation, active rTMS produced greater improvements in subjective and objective sleep outcomes, including reduced PSQI scores, increased sleep efficiency, and decreased wake after sleep onset and sleep latency. Active rTMS was also associated with greater longitudinal increases in global efficiency, local efficiency, clustering coefficient, and nodal efficiency in prefrontal, default-mode, temporal, and salience-related regions. Rich-club connectivity increased more strongly after active rTMS than after sham stimulation, whereas feeder connectivity, normalized rich-club coefficient, and local connectivity did not show significant group-by-time interaction effects after FDR correction. In the active rTMS group, increases in rich-club connectivity were positively associated with improvements in actigraphy-derived sleep efficiency (*r* = 0.43, *P* = 0.002).

**Conclusions:**

Active rTMS improved sleep quality more effectively than sham stimulation in patients with first-episode schizophrenia and was accompanied by longitudinal changes in DTI-derived white matter network organization. The association between increased rich-club connectivity and improved sleep efficiency suggests that hub-related network reorganization may represent a network-level correlate of rTMS-related sleep improvement.

## 1 Introduction

Sleep disturbances are among the most common non-psychotic symptoms in schizophrenia, affecting approximately 50–80% of patients and emerging early in the course of illness (Kahn, 2020). Beyond reducing quality of life, disturbed sleep has been associated with more severe psychotic symptoms, cognitive dysfunction, impaired social functioning, increased relapse risk, and poorer functional recovery (Bagautdinova et al., 2023; Ketcham et al., 2024; Wulff et al., 2012; Peng et al., 2024). Consequently, improving sleep disturbances has become an important therapeutic objective in the management of first-episode schizophrenia.

Repetitive transcranial magnetic stimulation (rTMS) is a non-invasive neuromodulation technique capable of modifying cortical excitability and large-scale brain network activity (Lanza et al., 2023). Growing evidence supports its therapeutic efficacy for several psychiatric disorders, including depression and schizophrenia (Cole et al., 2022; Stanford et al., 2008). More recently, randomized controlled studies and meta-analyses have suggested that rTMS may also improve both subjective and objective sleep outcomes in patients with insomnia (Ma et al., 2021). However, whether active rTMS provides clinically meaningful sleep improvement in first-episode schizophrenia remains largely unknown.

Emerging neuroimaging evidence indicates that sleep disturbances are accompanied by widespread alterations in brain structural connectivity rather than isolated regional abnormalities. Diffusion tensor imaging studies have consistently demonstrated disrupted white matter integrity and altered structural networks in individuals with chronic insomnia, involving fronto-limbic, thalamo-cortical, and salience systems (Sanjari Moghaddam et al., 2021). These observations suggest that successful treatment of sleep disturbances may involve large-scale structural network reorganization. Nevertheless, whether rTMS-related sleep improvement is accompanied by longitudinal alterations in white matter structural networks remains unknown.

Graph theoretical analysis provides a powerful framework for characterizing white matter structural networks beyond regional microstructural abnormalities (Kelly et al., 2018). Previous studies have consistently demonstrated disrupted structural network topology in schizophrenia, including reduced network efficiency, impaired information integration, and abnormal hub organization (Sanjari Moghaddam et al., 2021; Camchong et al., 2011). Recent genetic evidence further suggests a shared genetic architecture between schizophrenia and white matter microstructure, highlighting the biological relevance of DTI-derived white matter alterations in schizophrenia (Zhang et al., 2026). Among these network properties, rich-club organization represents a densely interconnected backbone supporting efficient global communication across the brain (van den Heuvel and Sporns, 2011; van den Heuvel et al., 2013). Alterations in rich-club connectivity have been reported in schizophrenia and may be particularly sensitive to neuromodulatory interventions (Guan et al., 2023).

Therefore, the present study investigated longitudinal changes in sleep and white matter structural networks following active or sham rTMS in patients with first-episode schizophrenia. We hypothesized that active rTMS would produce greater improvements in subjective and objective sleep than sham stimulation and that these improvements would be accompanied by longitudinal reorganization of white matter structural networks, particularly rich-club connectivity.

## 2 Materials and methods

### 2.1 Study design and participants

This study was designed as a randomized, sham-controlled, and longitudinal intervention study to investigate the effects of repetitive transcranial magnetic stimulation (rTMS) on sleep disturbances and white matter network organization in first-episode schizophrenia. A total of 120 patients with first-episode schizophrenia were assessed for eligibility. Ten participants were excluded before randomization because of declined participation, baseline MRI/DTI data quality issues, or incomplete baseline sleep assessment. The remaining 110 participants were randomly assigned in a 1:1 ratio to receive either active rTMS or sham rTMS. During the 8-week intervention, five participants in each group did not complete treatment or follow-up assessments. Finally, 100 participants were included in the final analysis, including 50 in the active rTMS group and 50 in the sham rTMS group. Given the limited availability of prior longitudinal studies providing effect-size estimates for rTMS-related changes in DTI-derived graph-theoretical network measures in first-episode schizophrenia, a formal a priori power calculation for these imaging outcomes was not performed. The study therefore recruited 120 patients to ensure an adequate sample for longitudinal imaging analyses while accounting for potential attrition and data loss. Longitudinal analyses were conducted in participants who completed both baseline and Week 8 assessments and were therefore based on the completer population rather than an intention-to-treat population.

Patients were recruited from Xijing Hospital. The diagnosis of schizophrenia was established by experienced psychiatrists according to the Diagnostic and Statistical Manual of Mental Disorders, Fifth Edition (DSM-5). First-episode schizophrenia was defined as the first lifetime episode of psychosis, with illness duration less than 6 months and no history of long-term antipsychotic treatment. For patients with an illness duration shorter than 6 months at enrollment, the diagnosis was confirmed after clinical follow-up. Antipsychotic medication dosage was converted into olanzapine equivalents according to the defined daily dose method (Leucht et al., 2016).

Inclusion criteria were as follows: age between 18 and 35 years; diagnosis of first-episode schizophrenia; right-handedness; normal or corrected-to-normal vision; ability to complete MRI and sleep assessments; and written informed consent. Exclusion criteria included a history of traumatic brain injury, neurological disorders, severe systemic illness, substance use disorder, contraindications to MRI or rTMS, current severe suicidal risk, and estimated intelligence quotient below 70. All participants received standard clinical care, including antipsychotic medication and psychosocial intervention, throughout the study period. Antipsychotic treatment was maintained as stable as clinically feasible during the 8-week intervention, and changes in medication type or dosage were avoided unless clinically necessary. Concomitant use of benzodiazepines and hypnotic medications during the intervention was reviewed to evaluate potential medication-related confounding of the sleep outcomes. The study was approved by the local Research Ethics Committee of Xijing Hospital and was conducted in accordance with the Declaration of Helsinki. All participants provided written informed consent before study procedures.

### 2.2 Randomization and blinding

Participants were randomly assigned to the active rTMS or sham rTMS group using a 1:1 allocation ratio. Randomization was performed using a computer-generated random sequence. The allocation sequence was concealed from participants, outcome assessors, and imaging analysts. Participants and outcome assessors were blinded to treatment allocation throughout the study. Sleep assessments, clinical evaluations, MRI acquisition, and image analyses were performed by investigators blinded to treatment allocation. Sham stimulation was delivered using a dedicated sham coil placed over the same target and according to the same treatment schedule as active rTMS. The sham coil was designed to mimic the acoustic and procedural characteristics of active stimulation while minimizing effective cortical stimulation. A formal assessment of blinding integrity by asking participants to guess their treatment allocation at the end of the intervention was not performed.

### 2.3 Clinical and sleep assessments

Clinical symptoms were assessed using the Positive and Negative Syndrome Scale (PANSS), including positive, negative, general psychopathology, and total scores. Demographic and clinical variables, including age, sex, education, illness duration, medication dosage, and head motion parameters, were recorded. Sleep quality was assessed using both subjective and objective measures at baseline and after the 8-week intervention. Subjective sleep quality was evaluated using the Pittsburgh Sleep Quality Index (PSQI). The PSQI total score ranges from 0 to 21, with higher scores indicating poorer sleep quality (Bergmann et al., 2021). Objective sleep parameters were obtained using wrist actigraphy. Participants wore actigraphy devices continuously during the sleep assessment period at baseline and again after the intervention. Actigraphy-derived measures included sleep efficiency, wake after sleep onset (WASO), and sleep latency. Sleep efficiency was calculated as the percentage of total sleep time relative to time in bed. The primary sleep outcomes were PSQI total score and actigraphy-derived sleep efficiency. Secondary sleep outcomes included WASO and sleep latency.

### 2.4 rTMS intervention

Repetitive transcranial magnetic stimulation (rTMS) was administered using a figure-eight focal coil connected to a MagPro 100 magnetic stimulator (MagVenture, Denmark). The resting motor threshold (RMT) was determined according to standard non-invasive brain stimulation guidelines (Guerra et al., 2015). The right dorsolateral pre-frontal cortex (DLPFC) was targeted individually using the F4 electrode position according to the international 10–20 EEG system (Mir-Moghtadaei et al., 2015). This standardized scalp-based targeting procedure was applied consistently across participants and throughout the intervention. Active rTMS was delivered at a frequency of 1 Hz with an intensity of 100% RMT. Each session consisted of 1,500 pulses. The intervention lasted 8 weeks. During weeks 1–4, participants received five sessions per week, followed by two sessions per week during weeks 5–8, resulting in a total of 28 sessions and 42,000 pulses over the treatment period.

Participants in the sham group received stimulation over the same target and according to the same schedule using a dedicated sham coil. The sham coil was designed to mimic the acoustic and procedural characteristics of active stimulation while minimizing effective cortical stimulation.

### 2.5 MRI data acquisition

MRI data were acquired at baseline and after the 8-week intervention using a 3.0-T Philips MRI scanner with a 32-channel head coil. Participants were instructed to remain still during scanning. Foam pads and earplugs were used to reduce head motion and scanner noise. Diffusion tensor imaging data were acquired using a single-shot echo-planar imaging sequence aligned to the anterior–posterior commissural plane. The acquisition parameters were as follows: field of view = 224 × 224 mm^2^, repetition time/echo time = 4,000/85 ms, flip angle = 90°, slice thickness = 2 mm, and 66 continuous axial slices. Diffusion-sensitizing gradients were applied along 64 non-collinear directions with a b value of 1,000 s/mm^2^, together with non-diffusion-weighted images with b = 0 s/mm^2^. High-resolution T1-weighted anatomical images were obtained using a three-dimensional magnetization-prepared rapid gradient-echo sequence. The parameters were as follows: repetition time = 8.2 ms, echo time = 3.18 ms, field of view = 256 × 256 mm^2^, matrix size = 256 × 256, slice thickness = 1 mm, and 210 continuous slices.

### 2.6 DTI preprocessing and tractography

DTI preprocessing was performed using ExploreDTI. Diffusion-weighted images were corrected for eddy-current distortions and head motion. Following correction, all diffusion datasets underwent quality control, including visual inspection for residual motion-related artifacts, signal dropout, and image distortion. Participants were excluded when substantial residual artifacts persisted after correction or when image quality was considered insufficient for reliable tensor estimation and whole-brain tractography.

Whole-brain deterministic tractography was performed using the Fiber Assignment by Continuous Tracking (FACT) algorithm. Tracking was initiated in voxels with FA > 0.20 and terminated when FA fell below 0.20 or when the turning angle between consecutive principal eigenvectors exceeded 45°. These thresholds were selected a priori based on established deterministic tractography procedures used in previous structural connectome studies (Ma et al., 2025; Guo et al., 2024), rather than being optimized specifically for the present dataset. The same tracking parameters were applied consistently across all participants and both imaging time points.

### 2.7 White matter network construction

Individual white matter structural networks were constructed using a graph-theoretical framework. Network nodes were defined according to the automated anatomical labeling atlas, which parcellates the brain into 90 cortical and subcortical regions excluding the cerebellum.

For each participant, the b0 image was linearly registered to the corresponding high-resolution T1-weighted image. The T1-weighted image was then nonlinearly registered to the Montreal Neurological Institute standard template. The inverse transformations were applied to warp the atlas from standard space into individual diffusion space. Each atlas region represented a network node.

Network edges were defined as white matter fiber connections between pairs of brain regions. The weight of each edge was defined by the number of reconstructed streamlines connecting the corresponding regions. To reduce false-positive connections, edges with fewer than 10 streamlines were set to zero. This procedure generated a weighted 90 × 90 white matter structural connectivity matrix for each participant at each time point.

### 2.8 Global and nodal network measures

Graph-theoretical analyses were performed on weighted white matter structural networks. Global network measures included global efficiency, local efficiency, clustering coefficient, shortest path length, and network strength. Global efficiency reflects the capacity for parallel information transfer across the whole network. Local efficiency reflects the fault tolerance and local information processing capacity of the network. The clustering coefficient quantifies the tendency of neighboring nodes to be interconnected. Shortest path length reflects the average minimum number of steps required for information transfer between nodes. Network strength was calculated as the sum of weighted connections across the network.

Nodal efficiency was calculated for each brain region to characterize regional contributions to information transfer within the structural network. Longitudinal changes in nodal efficiency were calculated as Week 8 minus baseline values.

### 2.9 Rich-club analysis

Rich-club organization was examined to characterize the connectivity among highly connected hub regions. For each group, a group-averaged backbone network was constructed based on significant non-zero connections across participants using a non-parametric one-tailed sign test. Nodes with a degree at least one standard deviation above the mean nodal degree were defined as rich-club regions.

Based on the classification of rich-club and peripheral nodes, structural connections were divided into three categories: rich-club connections, linking two rich-club nodes; feeder connections, linking rich-club nodes with peripheral nodes; and local connections, linking two peripheral nodes. The connection strength of each category was calculated as the sum of the corresponding edge weights.

The weighted rich-club coefficient was calculated as the ratio between the sum of weights among rich-club nodes and the sum of the strongest connections in the whole network with the same number of edges. Normalized rich-club coefficients were obtained by dividing the observed rich-club coefficient by the mean rich-club coefficient from 1,000 degree-preserving random networks. A normalized rich-club coefficient greater than 1 indicated the presence of rich-club organization.

Longitudinal changes in normalized rich-club coefficient, rich-club connectivity, feeder connectivity, and local connectivity were calculated as post-treatment minus baseline values.

### 2.10 Statistical analysis

Statistical analyses were performed using SPSS version 24.0 and appropriate statistical packages in MATLAB/R. Continuous variables are presented as mean ± standard deviation, and categorical variables are presented as counts. Baseline demographic and clinical characteristics were compared between the active and sham rTMS groups using independent-samples *t* tests for continuous variables and chi-square tests for categorical variables.

The primary treatment effects on sleep outcomes were assessed using linear mixed-effects models. Each model included group, time, and group-by-time interaction as fixed effects. The group-by-time interaction was the primary effect of interest, indicating whether longitudinal changes differed between active and sham rTMS groups. Age, sex, education, illness duration, medication dosage, and head motion were included as covariates where appropriate. Significant interaction effects were followed by *post hoc* within-group comparisons with correction for multiple comparisons.

For global network measures, longitudinal changes were analyzed using linear mixed-effects models with group, time, and group-by-time interaction as fixed effects. For nodal efficiency, group-by-time interaction effects were examined across all 90 brain regions. For rich-club analyses, group-by-time interaction effects were tested for the normalized rich-club coefficient, rich-club connectivity, feeder connectivity, and local connectivity.

To account for multiple testing across related outcomes, the Benjamini–Hochberg false discovery rate (FDR) procedure was applied separately within four conceptually defined families of measures: the four sleep outcomes (PSQI, sleep efficiency, WASO, and sleep latency), the four global network metrics (global efficiency, local efficiency, clustering coefficient, and shortest path length), nodal efficiency across the 90 brain regions, and the four rich-club measures (normalized rich-club coefficient, rich-club connectivity, feeder connectivity, and local connectivity). An FDR-adjusted *P* value (*q*) < 0.05 was considered statistically significant.

To examine the relationship between white matter network reorganization and sleep improvement, Pearson correlation analyses were performed between longitudinal changes in rich-club connectivity and changes in actigraphy-derived sleep efficiency in the active rTMS group. As a sensitivity analysis for potential motion-related confounding, correlations were additionally examined between longitudinal changes in head motion and changes in the principal network measures. Statistical significance was set at *P* < 0.05.

## 3 Results

### 3.1 Participant flow and baseline characteristics

A total of 120 patients with first-episode schizophrenia were assessed for eligibility. Ten participants were excluded before randomization because of declined participation, baseline MRI/DTI data quality issues, or incomplete baseline sleep assessment. The remaining 110 participants were randomly assigned to receive either active rTMS or sham rTMS. During the 8-week intervention, five participants in each group did not complete treatment or follow-up assessments. Finally, 100 participants were included in the final analysis, with 50 participants in the active rTMS group and 50 participants in the sham rTMS group (Figure 1).

**Figure 1:**
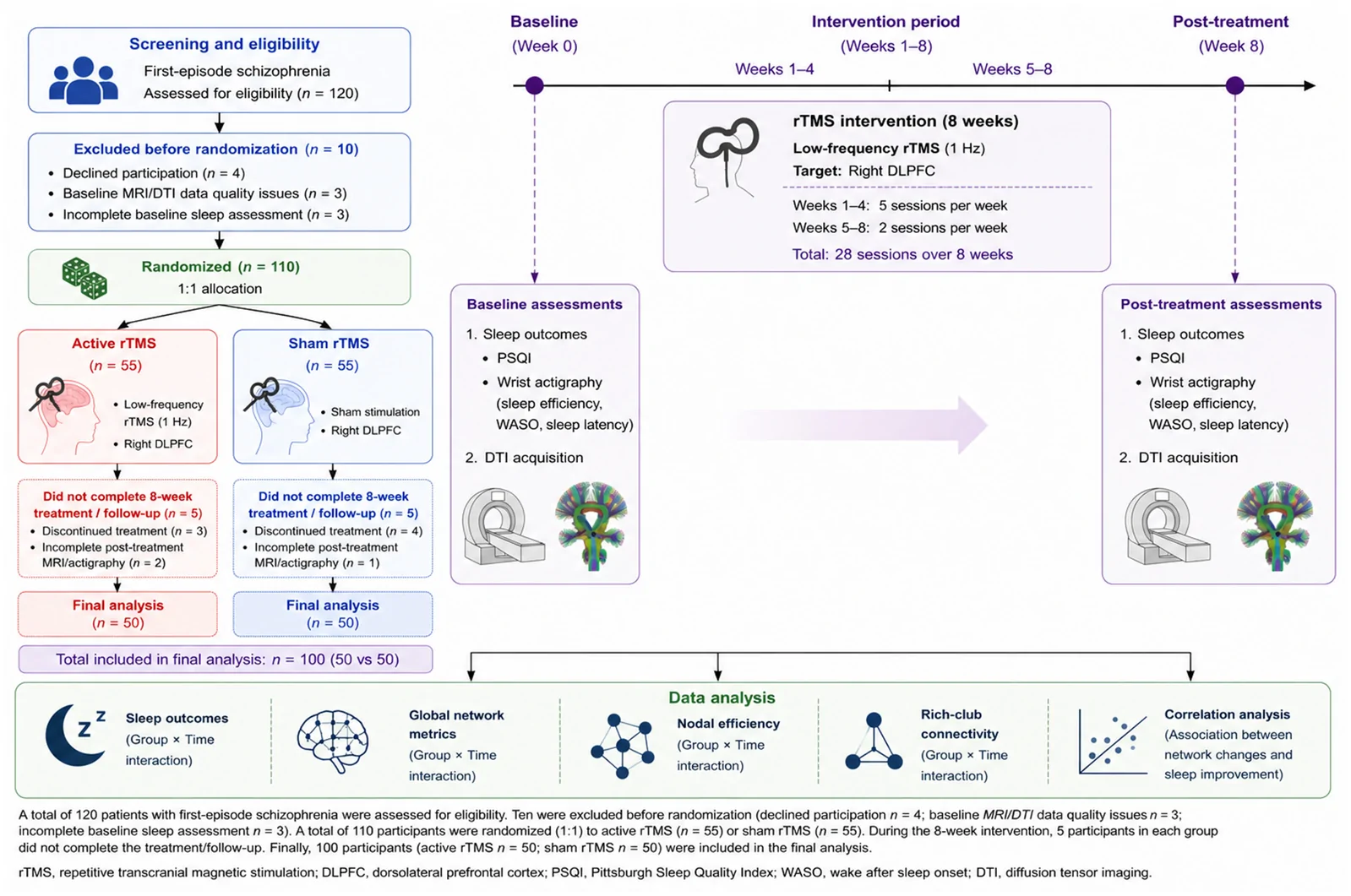
Study design and procedures. A total of 120 patients with first-episode schizophrenia were assessed for eligibility. Ten participants were excluded before randomization because of declined participation, baseline MRI/DTI data quality issues, or incomplete baseline sleep assessment. The remaining 110 participants were randomly assigned in a 1:1 ratio to receive either active rTMS or sham rTMS. During the 8-week intervention, five participants in each group did not complete treatment or follow-up, resulting in 100 participants included in the final analysis. Active rTMS consisted of low-frequency stimulation at 1 Hz over the right dorsolateral prefrontal cortex. Sleep assessments and DTI acquisition were performed at baseline and after the 8-week intervention. Data analyses included sleep outcomes, global network metrics, nodal efficiency, rich-club connectivity, and correlation analyses between network changes and sleep improvement. rTMS, repetitive transcranial magnetic stimulation; DLPFC, dorsolateral prefrontal cortex; PSQI, Pittsburgh Sleep Quality Index; WASO, wake after sleep onset; DTI, diffusion tensor imaging.

Baseline demographic and clinical characteristics were comparable between the active and sham rTMS groups. There were no significant between-group differences in age, sex, education, handedness, frame-wise displacement, duration of illness, olanzapine-equivalent antipsychotic dose, PANSS scores, or baseline sleep measures, including PSQI, sleep efficiency, WASO, and sleep latency (all *P* > 0.05; Table 1). To assess potential attrition-related bias, baseline demographic and clinical characteristics were also compared between participants who completed the study (*n* = 100) and those who did not complete the follow-up assessment (*n* = 10). No significant differences were observed between completers and non-completers in baseline demographic characteristics, illness duration, PANSS scores, or sleep measures (all *P* > 0.05). In addition, no significant between-group differences were observed in concomitant benzodiazepine or hypnotic medication use during the intervention (*P* > 0.05).

**Table 1:** Baseline demographic and clinical characteristics of participants in the active and sham rTMS groups.

Characteristic	Active rTMS (*n* = 50)	Sham rTMS (*n* = 50)	Statistical value	*P* value
Age: years	23.3 ± 3.7	23.7 ± 3.6	*t* = 0.55	0.584
Gender (male/female)	27/23	26/24	χ^2^ = 0.04	0.841
Education: years	12.5 ± 2.7	12.3 ± 2.6	*t* = 0.38	0.704
Handedness (right/left)	50/0	50/0	NA	NA
Frame-wise displacement	0.29 ± 0.09	0.28 ± 0.08	*t* = 0.59	0.556
Duration of illness: month	2.9 ± 2.1	3.1 ± 2.0	*t* = 0.49	0.626
PANSS scores				
Positive score	22.4 ± 7.2	22.8 ± 6.9	*t* = 0.29	0.771
Negative score	22.8 ± 6.1	22.4 ± 6.4	*t* = 0.32	0.748
General score	47.3 ± 7.9	47.8 ± 8.0	*t* = 0.31	0.756
Total score	87.8 ± 16.6	88.4 ± 16.1	*t* = 0.18	0.857
Sleep measures				
PSQI	8.2 ± 1.4	8.3 ± 1.5	*t* = 0.34	0.737
Sleep Efficiency	75.4 ± 4.7	75.1 ± 4.9	*t* = 0.31	0.758
WASO	56.7 ± 11.4	57.1 ± 11.0	*t* = 0.18	0.86
Sleep latency	47.3 ± 11.5	46.8 ± 11.6	*t* = 0.22	0.824

Data are presented as mean ± standard deviation or number of participants, as appropriate. Between-group differences at baseline were assessed using independent-samples *t* tests for continuous variables and chi-square tests for categorical variables. rTMS, repetitive transcranial magnetic stimulation; PANSS, Positive and Negative Syndrome Scale; PSQI, Pittsburgh Sleep Quality Index; WASO, wake after sleep onset; NA, not applicable.

### 3.2 Active rTMS produced greater improvements in subjective and objective sleep outcomes

Linear mixed-effects models revealed significant group-by-time interaction effects for all sleep outcomes, indicating that active rTMS produced greater sleep improvement than sham stimulation. All four interaction effects remained significant after FDR correction across the sleep outcome family (all FDR-adjusted *q* < 0.001).

For subjective sleep quality, PSQI scores decreased from 8.2 ± 1.4 at baseline to 5.1 ± 1.6 at Week 8 in the active rTMS group, whereas the sham group showed only a modest change from 8.3 ± 1.5 to 7.8 ± 1.6. The corresponding mean reduction in PSQI score was 3.1 points in the active rTMS group, compared with 0.5 points in the sham group. The group-by-time interaction was significant (*raw P* < 0.001; FDR-adjusted *q* < 0.001; Table 2).

**Table 2:** Longitudinal changes in sleep outcomes following active versus sham rTMS.

Sleep measure	Active rTMS baseline	Active rTMS week 8	Active rTMS Δ change	Sham rTMS baseline	Sham rTMS week 8	Sham rTMS Δ change	Group × Time *P* value
PSQI	8.2 ± 1.4	5.1 ± 1.6^***^	−3.1	8.3 ± 1.5	7.8 ± 1.6	−0.5	< 0.001
Sleep efficiency (%)	75.4 ± 4.7	79.5 ± 5.4^***^	+4.1	75.1 ± 4.9	75.9 ± 5.0	+0.8	< 0.001
WASO (min)	56.7 ± 11.4	42.6 ± 10.0^***^	−14.1	57.1 ± 11.0	55.4 ± 11.3	−1.7	< 0.001
Sleep latency (min)	47.3 ± 11.5	30.3 ± 9.6^***^	−17.0	46.8 ± 11.6	44.9 ± 11.4	−1.9	< 0.001

Data are presented as mean ± standard deviation unless otherwise indicated. Δ Change was calculated as Week 8 minus baseline; values are shown as mean changes. Longitudinal effects were assessed using linear mixed-effects models with group, time, and group-by-time interaction as fixed effects. Significant group-by-time interactions were followed by *post hoc* within-group comparisons with Bonferroni correction. ^***^P < 0.001 vs. baseline within the active rTMS group. PSQI, Pittsburgh Sleep Quality Index; WASO, wake after sleep onset; rTMS, repetitive transcranial magnetic stimulation.

For objective sleep measures derived from actigraphy, sleep efficiency increased from 75.4 ± 4.7% to 79.5 ± 5.4% in the active rTMS group, while the sham group showed a smaller increase from 75.1 ± 4.9% to 75.9 ± 5.0% (*raw P* < 0.001; FDR-adjusted *q* < 0.001; Table 2). WASO decreased from 56.7 ± 11.4 min to 42.6 ± 10.0 min after active rTMS, compared with a smaller decrease from 57.1 ± 11.0 min to 55.4 ± 11.3 min in the sham group (*raw P* < 0.001; FDR-adjusted *q* < 0.001; Table 2). Sleep latency also decreased more markedly in the active rTMS group, from 47.3 ± 11.5 min to 30.3 ± 9.6 min, compared with 46.8 ± 11.6 min to 44.9 ± 11.4 min in the sham group (*raw P* < 0.001; FDR-adjusted *q* < 0.001; Table 2). These findings indicate that active rTMS improved both subjective and objective sleep parameters more effectively than sham stimulation. To determine whether the greater sleep improvement following active rTMS could be attributable to differential changes in psychiatric symptom severity, longitudinal changes in PANSS scores were also examined. Both groups showed significant reductions in PANSS positive, general psychopathology, and total scores over the 8-week treatment period, whereas changes in PANSS negative scores were not significant. However, no significant group-by-time interactions were observed for PANSS positive, negative, general psychopathology, or total scores (all *P* > 0.4; Table 2), indicating comparable changes in psychiatric symptoms between the active and sham rTMS groups.

### 3.3 Active rTMS was associated with greater longitudinal changes in global white matter network topology

Longitudinal changes in global white matter network measures were compared between the active and sham rTMS groups (Figure 2). After FDR correction across the four global network metrics, significant group-by-time interactions remained for global efficiency, local efficiency, and clustering coefficient, whereas shortest path length remained non-significant.

**Figure 2:**
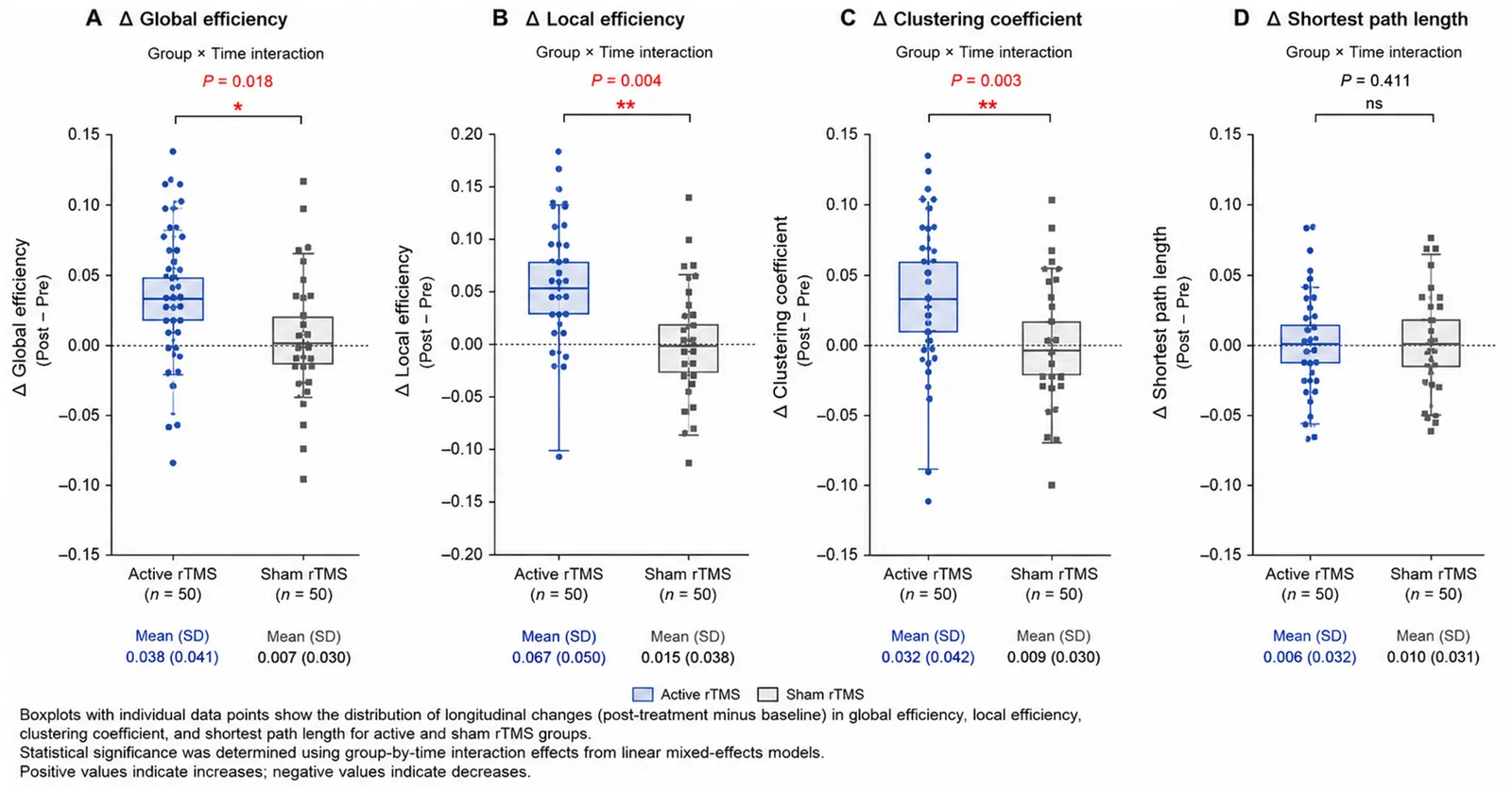
Longitudinal changes in global white matter network topology following active vs. sham rTMS. Boxplots with individual data points show longitudinal changes in global white matter network metrics, including global efficiency, local efficiency, clustering coefficient, and shortest path length. Δ values were calculated as post-treatment minus baseline. Group-by-time interaction effects were assessed using linear mixed-effects models. Active rTMS was associated with greater increases in global efficiency, local efficiency, and clustering coefficient compared with sham stimulation, whereas no significant group-by-time interaction was observed for shortest path length. Positive values indicate increases after treatment, whereas negative values indicate decreases.

The increase in global efficiency was greater in the active rTMS group than in the sham group (ΔActive = 0.038 ± 0.041; ΔSham = 0.007 ± 0.030; group-by-time interaction, *raw P* = 0.018, FDR-adjusted *q* = 0.024). Similarly, local efficiency showed a greater increase following active rTMS than sham stimulation (ΔActive = 0.067 ± 0.050; ΔSham = 0.015 ± 0.038; *raw P* = 0.004, FDR-adjusted *q* = 0.008). The clustering coefficient also increased more strongly in the active group (ΔActive = 0.032 ± 0.042) than in the sham group (ΔSham = 0.009 ± 0.030; *raw P* = 0.003, FDR-adjusted *q* = 0.008). In contrast, no significant group-by-time interaction was observed for shortest path length (ΔActive = 0.006 ± 0.032; ΔSham = 0.010 ± 0.031; *raw P* = 0.411, FDR-adjusted *q* = 0.411).

These findings suggest that active rTMS was associated with enhanced global and local integration of the white matter structural network, while shortest path length remained relatively unchanged.

### 3.4 Active rTMS induced greater longitudinal increases in nodal efficiency

We next examined nodal efficiency to identify regional white matter network changes associated with active rTMS. After FDR correction across the 90 brain regions, significant group-by-time interaction effects were observed in six regions: the left dorsolateral superior frontal gyrus, left precuneus, left posterior cingulate gyrus, right dorsolateral superior frontal gyrus, right middle temporal gyrus, and left insula (Figure 3; Table 3).

**Figure 3:**
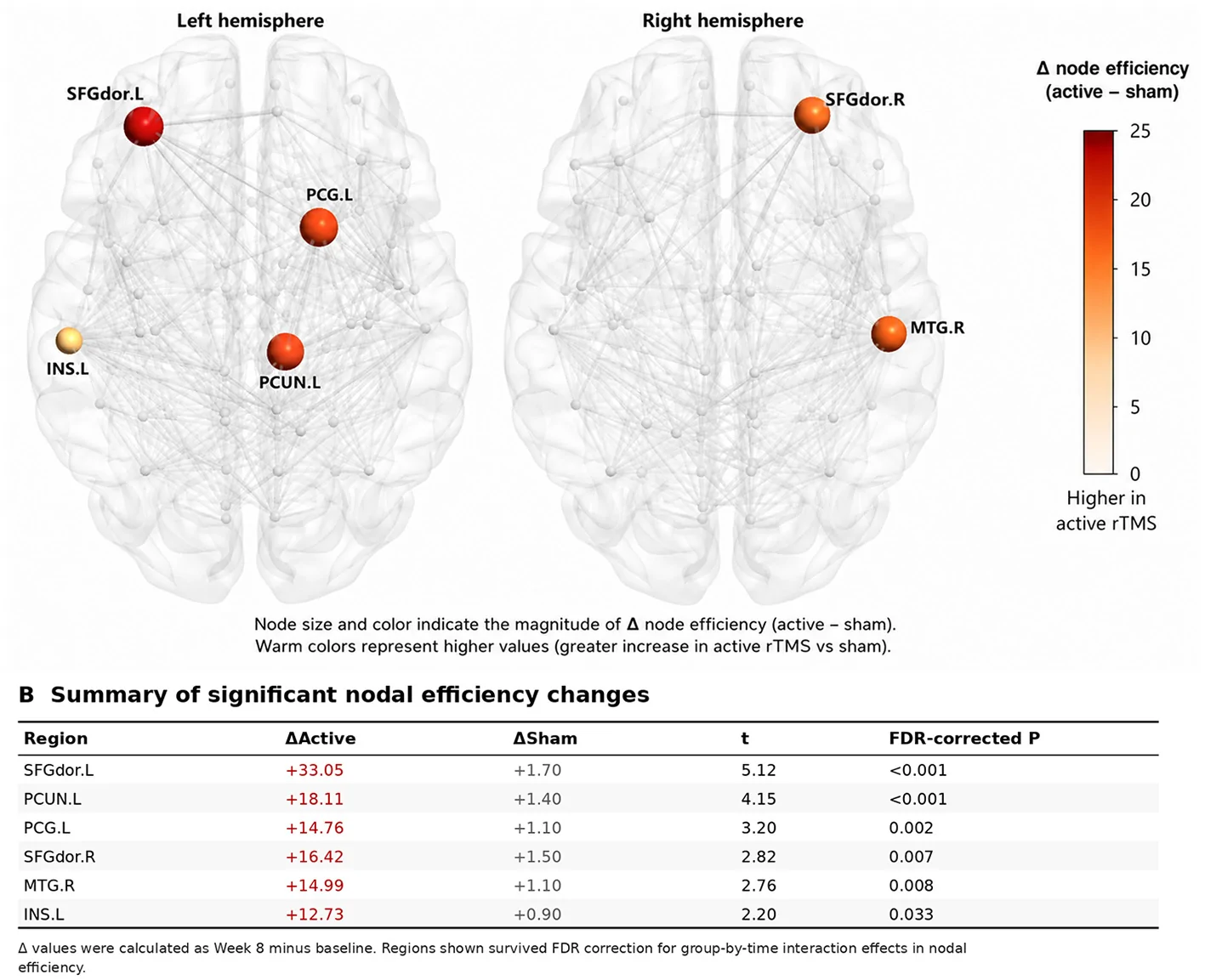
Brain regions exhibiting greater longitudinal increases in nodal efficiency following active relative to sham rTMS. **(A)** Brain regions showing significant group-by-time interaction effects in nodal efficiency are displayed on the white matter structural network. Red spheres indicate regions with greater longitudinal increases in nodal efficiency in the active rTMS group compared with the sham rTMS group after false discovery rate correction. Node size and color represent the magnitude of the between-group difference in longitudinal change, with warmer colors indicating greater increases following active rTMS. **(B)** Summary of significant regions showing group-by-time interaction effects in nodal efficiency. ΔActive and ΔSham values were calculated as Week 8 minus baseline within each group. Statistical values and FDR-corrected *P* values are reported for the between-group comparison of longitudinal changes. SFGdor, superior frontal gyrus, dorsolateral; PCUN, precuneus; PCG, posterior cingulate gyrus; MTG, middle temporal gyrus; INS, insula; L, left; R, right; rTMS, repetitive transcranial magnetic stimulation; FDR, false discovery rate.

**Table 3:** Brain regions showing significant group-by-time interaction effects in nodal efficiency following active versus sham rTMS.

Regions	Active baseline	Active week 8	Delta active	Sham baseline	Sham week 8	Delta Sham	Statistical value
SFGdor.L	199.42 ± 23.94	232.47 ± 39.54	+33.05	200.60 ± 24.80	202.30 ± 25.60	+1.70	*t* = 5.12
PCUN.L	184.91 ± 20.57	203.02 ± 26.25	+18.11	185.60 ± 21.30	187.00 ± 21.70	+1.40	*t* = 4.15
PCG.L	173.68 ± 18.67	188.44 ± 32.24	+14.76	174.50 ± 19.40	175.60 ± 20.00	+1.10	*t* = 3.20
SFGdor.R	202.26 ± 31.43	218.68 ± 27.77	+16.42	201.10 ± 30.20	202.60 ± 29.80	+1.50	*t* = 2.82
MTG.R	195.06 ± 26.09	210.05 ± 32.35	+14.99	195.80 ± 25.70	196.90 ± 26.40	+1.10	*t* = 2.76
INS.L	173.26 ± 21.37	185.99 ± 43.70	+12.73	174.10 ± 22.40	175.00 ± 23.10	+0.90	*t* = 2.20

Data are presented as mean ± standard deviation. Delta values were calculated as Week 8 minus baseline. Nodal efficiency was analyzed using linear mixed-effects models with group, time, and group-by-time interaction as fixed effects. Brain regions listed in the table showed significant group-by-time interaction effects after false discovery rate correction. SFGdor, superior frontal gyrus, dorsolateral; PCUN, precuneus; PCG, posterior cingulate gyrus; MTG, middle temporal gyrus; INS, insula; L, left; R, right; rTMS, repetitive transcranial magnetic stimulation.

The largest effect was observed in the left dorsolateral superior frontal gyrus, where nodal efficiency increased substantially in the active rTMS group but only minimally in the sham group (ΔActive = +33.05; ΔSham = +1.70; *t* = 5.12; FDR-adjusted *q* < 0.001). Similar interaction effects were observed in the left precuneus (ΔActive = +18.11; ΔSham = +1.40; *t* = 4.15; FDR-adjusted *q* < 0.001), left posterior cingulate gyrus (ΔActive = +14.76; ΔSham = +1.10; *t* = 3.20; FDR-adjusted *q* = 0.002), right dorsolateral superior frontal gyrus (ΔActive = +16.42; ΔSham = +1.50; *t* = 2.82; FDR-adjusted *q* = 0.007), right middle temporal gyrus (ΔActive = +14.99; ΔSham = +1.10; *t* = 2.76; FDR-adjusted *q* = 0.008), and left insula (ΔActive = +12.73; ΔSham = +0.90; *t* = 2.20; FDR-adjusted *q* = 0.033).

These findings indicate that active rTMS produced greater regional increases in nodal efficiency within frontal, default-mode, temporal, and salience-related regions.

### 3.5 Active rTMS was associated with increased rich-club connectivity

Rich-club organization was further examined to determine whether active rTMS preferentially altered hub-related structural connectivity (Figure 4). After FDR correction across the four rich-club measures, a significant group-by-time interaction remained for rich-club connectivity, whereas the effects for normalized rich-club coefficient, feeder connectivity, and local connectivity were not significant.

**Figure 4:**
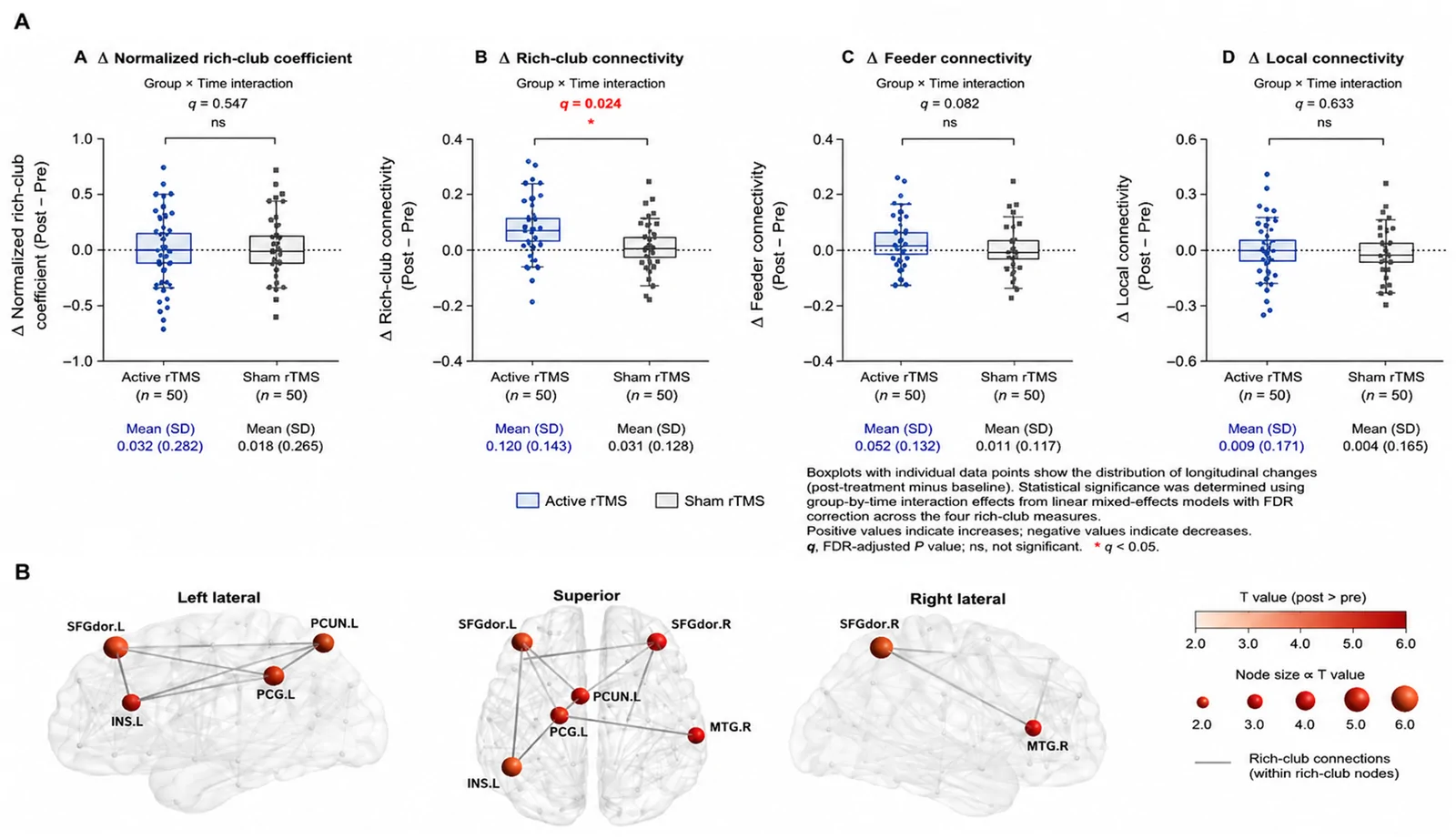
Longitudinal changes in rich-club organization following active vs. sham rTMS. **(A)** Longitudinal changes in the normalized rich-club coefficient, rich-club connectivity, feeder connectivity, and local connectivity in the active and sham rTMS groups. Boxplots show individual longitudinal changes (post-treatment minus baseline). Statistical significance was assessed using group-by-time interaction effects from linear mixed-effects models, with false discovery rate (FDR) correction applied across the four rich-club measures. After FDR correction, the group-by-time interaction remained significant for rich-club connectivity (*q* = 0.024), whereas feeder connectivity did not survive correction (*q* = 0.082). The normalized rich-club coefficient (*q* = 0.547) and local connectivity (*q* = 0.633) were not significant. **(B)** Visualization of the rich-club network showing greater longitudinal increases in connectivity following active rTMS. Red nodes represent significant rich-club regions, and gray lines indicate rich-club connections among these nodes. Node size and color are proportional to the corresponding *T* values, with warmer colors indicating stronger longitudinal effects.

No significant group-by-time interaction was observed for the normalized rich-club coefficient (ΔActive = 0.032 ± 0.282; ΔSham = 0.018 ± 0.265; *raw P* = 0.410, FDR-adjusted *q* = 0.547), indicating that the overall presence of rich-club organization remained relatively stable. Active rTMS was associated with a significantly greater increase in rich-club connectivity compared with sham stimulation (ΔActive = 0.120 ± 0.143; ΔSham = 0.031 ± 0.128; *raw P* = 0.006, FDR-adjusted *q* = 0.024). Feeder connectivity showed a nominal group-by-time effect before correction (ΔActive = 0.052 ± 0.132; ΔSham = 0.011 ± 0.117; *raw P* = 0.041), but this effect did not survive FDR correction (*q* = 0.082). Changes in local connectivity did not differ significantly between groups (ΔActive = 0.009 ± 0.171; ΔSham = 0.004 ± 0.165; *raw P* = 0.633, FDR-adjusted *q* = 0.633).

Visualization of the rich-club network showed increased connectivity among key hub-related regions following active rTMS, including the bilateral dorsolateral superior frontal gyrus, left precuneus, left posterior cingulate gyrus, left insula, and right middle temporal gyrus (Figure 4B). These results suggest that the most robust hub-related effect of active rTMS was an increase in rich-club connectivity, rather than a generalized increase across all connection classes. As a sensitivity analysis for potential motion-related confounding, longitudinal changes in head motion were not significantly correlated with changes in the principal network measures, including global efficiency, local efficiency, clustering coefficient, and rich-club connectivity (all *P* > 0.05). These findings provided no evidence that the observed longitudinal network changes were systematically related to changes in head motion.

### 3.6 Changes in rich-club connectivity were associated with improvements in sleep efficiency

Finally, we examined whether white matter network reorganization was associated with sleep improvement in the active rTMS group. Pearson correlation analysis showed that longitudinal increases in rich-club connectivity were positively associated with improvements in actigraphy-derived sleep efficiency (*r* = 0.43, *P* = 0.002; Figure 5). Participants with greater increases in rich-club connectivity tended to show larger improvements in sleep efficiency after the 8-week active rTMS intervention. This association suggests that changes in rich-club connectivity may represent a network-level correlate of rTMS-related sleep improvement in first-episode schizophrenia.

**Figure 5:**
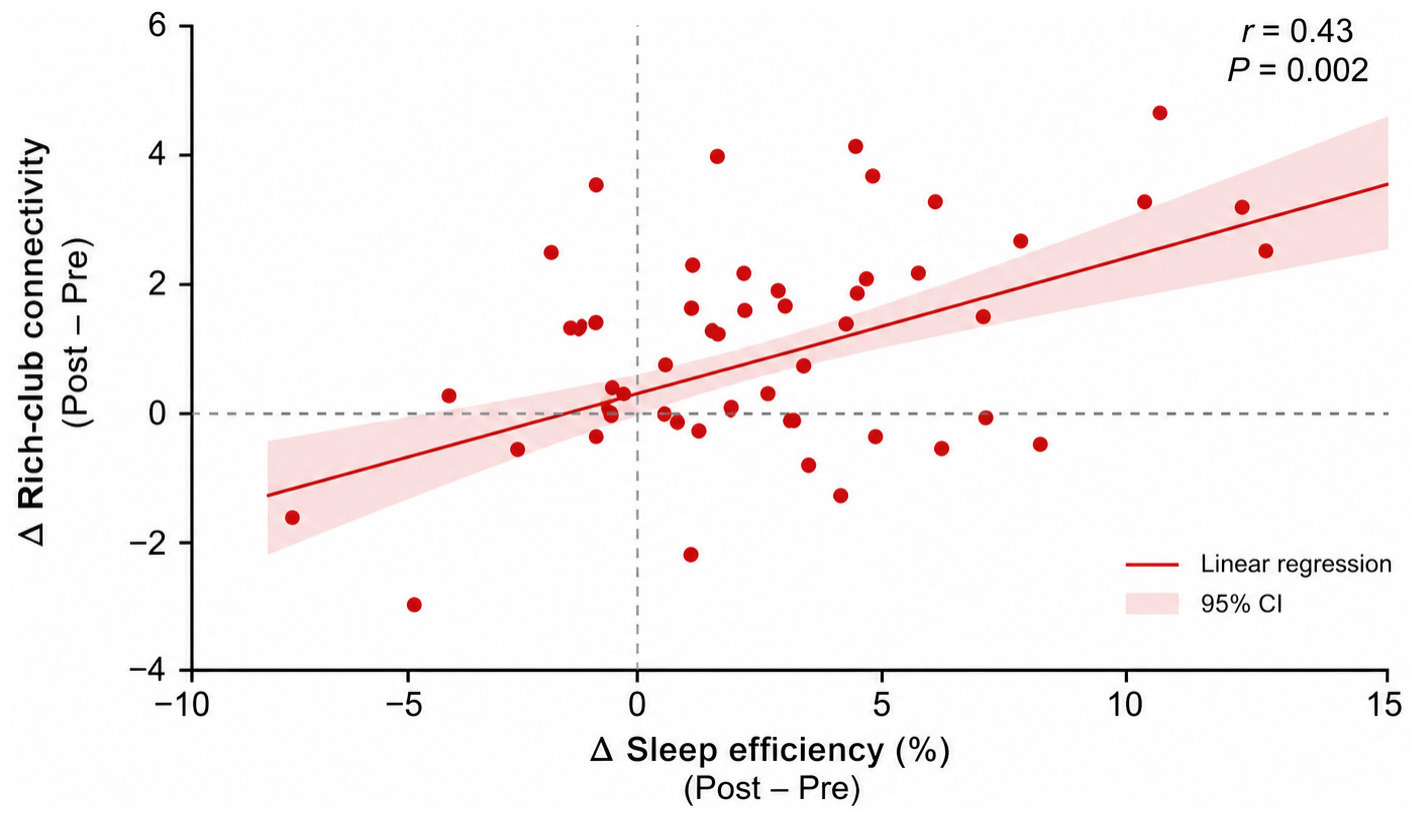
Association between longitudinal changes in rich-club connectivity and sleep efficiency following active rTMS. Scatter plot showing the association between changes in rich-club connectivity and changes in actigraphy-derived sleep efficiency in the active rTMS group. Δ values were calculated as post-treatment minus baseline. Each point represents one participant. The solid line indicates the least-squares regression line, and the shaded area represents the 95% confidence interval. Pearson's correlation coefficient and corresponding *P* value are shown in the figure. A positive association indicates that greater increases in rich-club connectivity were related to greater improvements in sleep efficiency.

## 4 Discussion

In this randomized sham-controlled longitudinal study, we investigated whether active rTMS improved sleep disturbances in first-episode schizophrenia and whether treatment-related sleep improvement was associated with DTI-derived white matter network reorganization. There were three principal findings. First, active rTMS produced greater improvements in both subjective and objective sleep outcomes than sham stimulation, including lower PSQI scores, higher actigraphy-derived sleep efficiency, and reductions in WASO and sleep latency. Second, active rTMS was associated with longitudinal changes in white matter structural network topology, reflected by greater increases in global efficiency, local efficiency, clustering coefficient, and regional nodal efficiency. Third, increases in rich-club connectivity were positively associated with improvements in sleep efficiency, suggesting an association between hub-related DTI-derived network changes and rTMS-related sleep improvement in first-episode schizophrenia.

Sleep disturbances are increasingly recognized as clinically meaningful treatment targets in schizophrenia and early psychosis. Prior studies have shown that sleep abnormalities are common across psychosis stages and are associated with symptom severity, cognitive dysfunction, suicidal ideation, and poorer functional outcomes (Bagautdinova et al., 2023; Zhao et al., 2026). In the present study, active rTMS led to significant improvements in both PSQI and actigraphy-derived sleep indices compared with sham stimulation. Although a schizophrenia-specific minimal clinically important difference (MCID) for the PSQI has not been firmly established, previous insomnia intervention studies have used reductions of approximately 2.5–3 points to represent minimally important or clinically meaningful change (Qin et al., 2024). In the present study, the PSQI score decreased by 3.1 points in the active rTMS group (from 8.2 to 5.1), compared with a 0.5-point reduction in the sham group. Thus, while this change cannot be interpreted against a validated schizophrenia-specific MCID, its magnitude is comparable to thresholds previously used to indicate clinically meaningful improvement in insomnia studies. Importantly, the improvement in subjective sleep quality was accompanied by significant improvements in actigraphy-derived sleep efficiency, WASO, and sleep latency, providing convergent support for the clinical relevance of the observed sleep changes. Notably, although both groups showed improvements in positive symptoms, general psychopathology, and overall symptom severity, no significant group-by-time interactions were observed for any PANSS measure (all *P* > 0.4). This pattern suggests that the greater improvement in sleep outcomes following active rTMS was unlikely to be solely attributable to greater overall improvement in psychiatric symptoms. These findings extend previous evidence that rTMS may improve insomnia symptoms in non-schizophrenia populations (Lanza et al., 2023; Ma et al., 2021; Sun et al., 2021) and suggest that low-frequency right DLPFC stimulation may also be beneficial for sleep disturbances in first-episode schizophrenia.

The observed sleep improvement may be related to the neuromodulatory effects of low-frequency rTMS on pre-frontal cortical excitability and large-scale network regulation. Insomnia has often been conceptualized within a hyperarousal framework, involving excessive cognitive, emotional, and physiological activation. Low-frequency rTMS is generally considered to reduce cortical excitability at the stimulated site, and stimulation of pre-frontal regions may modulate top-down control over arousal-related systems (Zhang S. et al., 2025; Zhang H. et al., 2025). Importantly, however, local inhibitory effects should not be equated with global suppression of network function. A systematic review of resting-state connectivity studies has shown that rTMS can induce changes both proximal and distal to the stimulation site and that the conventional low-frequency-inhibitory/high-frequency-facilitatory model does not fully account for distal network effects (Beynel et al., 2020). The DLPFC is embedded within distributed prefrontal, limbic, default-mode, and arousal-related circuits, and repeated modulation of this region may propagate through anatomically and functionally interconnected pathways, thereby altering network-level organization beyond the stimulation site. Moreover, the propagation of TMS effects across distant brain regions is related to macroscale structural connectivity, supporting the concept that focal stimulation can influence interconnected network nodes (Momi et al., 2021). From this perspective, the increased nodal efficiency and rich-club connectivity observed after active rTMS are not necessarily inconsistent with the inhibitory nature of 1-Hz stimulation, but may reflect distributed network reorganization following repeated modulation of the right DLPFC. In schizophrenia, where pre-frontal dysfunction and impaired network integration are prominent neurobiological features, such network-level effects may contribute to more coordinated communication across regions involved in arousal regulation, salience processing, and sleep–wake stability. However, because the present study did not directly measure cortical excitability or the temporal propagation of stimulation effects, this mechanistic interpretation remains tentative and requires further validation. The right DLPFC was localized using the F4 position of the international 10–20 EEG system, a standardized scalp-based approach with established anatomical correspondence to prefrontal cortical regions (Herwig et al., 2003). Although MRI-guided neuronavigation can provide greater individualized anatomical precision, this greater spatial precision has not consistently been shown to translate into superior clinical outcomes compared with EEG-based DLPFC targeting (Hebel et al., 2021). Nevertheless, interindividual variability in cortical anatomy may introduce variability in the effective stimulation site and should be considered when interpreting the present findings.

Beyond sleep improvement, active rTMS was associated with changes in global white matter network topology. Specifically, global efficiency, local efficiency, and clustering coefficient increased more strongly in the active rTMS group than in the sham group, whereas shortest path length did not show a significant group-by-time interaction. These findings suggest that rTMS-related effects may involve enhanced network integration and local information processing rather than a global shortening of communication paths. Structural connectivity abnormalities are well documented in schizophrenia, including widespread white matter microstructural disruptions and altered network organization (Kelly et al., 2018; Camchong et al., 2011; Yan et al., 2024). Our longitudinal findings build on this literature by suggesting that DTI-derived white matter network topology may be sensitive to neuromodulatory intervention in the early stage of schizophrenia. Importantly, these longitudinal changes should not be interpreted as direct evidence of axonal remodeling, myelination, or structural “repair” over the 8-week intervention period. DTI and tractography provide indirect measures of white matter organization rather than direct histological measures of axonal or myelin structure. Changes in diffusion-derived network properties may therefore arise from multiple underlying processes, including alterations in extracellular water diffusion, tissue microenvironment, and neuroinflammatory status, as well as changes in white matter microstructural organization. In addition, diffusion-derived network measures may be influenced by acquisition, preprocessing, and tractography-related factors. Accordingly, the present findings are more appropriately interpreted as longitudinal reorganization of DTI-derived white matter network properties rather than direct evidence of structural remodeling. Multimodal and advanced diffusion imaging approaches will be required to clarify the biological processes underlying these network changes.

At the regional level, active rTMS induced greater increases in nodal efficiency in the bilateral dorsolateral superior frontal gyrus, left precuneus, left posterior cingulate gyrus, right middle temporal gyrus, and left insula. These regions are relevant to the functional systems implicated in schizophrenia and sleep regulation. The dorsolateral superior frontal gyrus is closely related to the stimulation target and may reflect local or network-level consequences of prefrontal neuromodulation. The precuneus and posterior cingulate are core regions of the default-mode network, which has been implicated in self-referential processing, internally directed cognition, and sleep-related changes in brain organization. The insula is a key component of the salience network and is involved in interoceptive awareness and arousal regulation, while temporal regions may participate in perceptual and emotional processing. The convergence of nodal changes across prefrontal, default-mode, salience, and temporal regions further supports the possibility that rTMS-related effects extend beyond the local stimulation site and involve distributed network reorganization (Pei et al., 2025).

A central finding of this study was the selective increase in rich-club connectivity following active rTMS. Rich-club organization refers to a backbone of highly connected hub regions that supports efficient global communication across the brain (van den Heuvel et al., 2013; Kim and Min, 2020). Abnormal rich-club connectivity has been reported in schizophrenia and may contribute to inefficient integration among distributed neural systems (van den Heuvel et al., 2013). More recently, rich-club reorganization of white matter structural networks has been observed following 1 Hz rTMS in schizophrenia patients with auditory verbal hallucinations, suggesting that hub-related structural connectivity may be sensitive to neuromodulatory intervention (Guan et al., 2023). In the present study, the increase in rich-club connectivity remained significant after FDR correction, whereas the effect on feeder connectivity did not survive correction. No significant effects were observed for the normalized rich-club coefficient or local connectivity. This pattern suggests that rTMS may preferentially increase DTI-derived connectivity among existing hub regions, rather than fundamentally changing the overall presence of rich-club organization or producing a generalized increase across all connection classes. Such hub-focused network reorganization may be particularly relevant for sleep improvement, given that sleep depends on coordinated communication across distributed arousal, cognitive control, and internally oriented networks (Liu et al., 2025; Sun et al., 2025).

The positive association between changes in rich-club connectivity and improvements in sleep efficiency supports a link between DTI-derived network changes and sleep improvement. Participants who showed greater increases in rich-club connectivity tended to exhibit larger improvements in actigraphy-derived sleep efficiency. This finding is consistent with the view that rich-club architecture provides a central communication backbone for large-scale brain integration (van den Heuvel and Sporns, 2011; van den Heuvel et al., 2013). However, this association does not establish causality or temporal directionality. The present two-time-point design cannot determine whether DTI-derived network changes precede sleep improvement, whether improved sleep contributes to subsequent changes in diffusion-related network properties, or whether both represent parallel responses to rTMS. Future studies incorporating multiple intermediate assessment time points and multimodal imaging will be needed to establish temporal precedence and determine the direction of this relationship.

This study has several strengths. First, the randomized sham-controlled design improves causal inference compared with uncontrolled pre-post designs. Second, the use of a dedicated sham coil reduces expectancy and procedural confounds and strengthens the interpretation of active stimulation effects. Third, sleep outcomes were assessed using both PSQI and wrist actigraphy, allowing evaluation of both subjective sleep quality and objective sleep parameters. Fourth, the longitudinal DTI design enabled examination of treatment-related changes in global network topology, regional nodal efficiency, and rich-club connectivity, providing a multi-level characterization of DTI-derived white matter network changes.

Several limitations should be considered when interpreting the present findings. First, although wrist actigraphy does not directly characterize sleep architecture, it enabled continuous multi-night assessment of habitual sleep in the participants' natural environment, and the actigraphy-derived changes were consistent with the improvement in subjective sleep quality. Future studies incorporating polysomnography may further determine whether rTMS influences specific sleep stages. Second, although medication dosage was considered in the analyses and antipsychotic treatment was maintained as stable as clinically feasible, residual effects related to individual psychotropic agents cannot be entirely excluded. Third, deterministic tractography has limited ability to resolve complex fiber configurations, such as crossing or kissing fibers, and network estimates may depend partly on the selected parcellation scheme, including the exclusion of the cerebellum in the AAL90 atlas. Nevertheless, identical reconstruction procedures were applied across groups and time points, supporting the internal consistency of the longitudinal comparisons. Fourth, although blinding integrity was not formally quantified by post-intervention allocation guesses, a dedicated sham coil was used, and the concordant changes in subjective and actigraphy-derived sleep outcomes provide complementary support for the observed treatment effect. In addition, because the study was initially designed primarily as a longitudinal neuroimaging investigation of rTMS-related network changes rather than as a conventional clinical efficacy trial, it was not prospectively registered in a public clinical trial registry. Nevertheless, the study protocol was established and received institutional ethics approval before participant enrollment. Finally, the association between DTI-derived network changes and sleep improvement should not be interpreted as evidence of directionality or causality. Future studies incorporating multiple longitudinal assessment time points will be needed to clarify the temporal relationship between network reorganization and sleep improvement.

In conclusion, active rTMS produced greater improvements in subjective and objective sleep outcomes than sham stimulation in first-episode schizophrenia. These improvements were accompanied by longitudinal changes in DTI-derived white matter network organization, including increased global and local network efficiency, enhanced nodal efficiency in pre-frontal and hub-related regions, and increased rich-club connectivity. The association between changes in rich-club connectivity and improvements in sleep efficiency suggests that hub-related DTI-derived network reorganization may represent a network-level correlate of rTMS-related sleep improvement. These findings support the potential value of rTMS as a neuromodulatory approach for sleep disturbances in first-episode schizophrenia and identify longitudinal changes in DTI-derived white matter network organization as a potential neural correlate warranting further investigation.

## Data Availability

The raw data supporting the conclusions of this article will be made available by the authors, without undue reservation.
